# Supplementary material for: The formation of KV2.1 macro-clusters is required for sex-specific differences in L-type CaV1.2 clustering and function in arterial myocytes
Source: Commun Biol. 2023 Nov 14;6:1165. doi: 10.1038/s42003-023-05527-1 (PMC10645748; doi:10.1038/s42003-023-05527-1)
Supplement: Supplementary file 1 — Supplementary Information [file 42003_2023_5527_MOESM1_ESM.pdf]

Supplementary Materials for

# The formation of $K_V2.1$ macro-clusters is required for sex-specific differences in L-type $Ca_V1.2$ clustering and function in arterial myocytes

Collin Matsumoto[1], Samantha C. O'Dwyer[1], Declan Manning[1], Gonzalo Hernandez-Hernandez[1], Paula Rhana[1], Zhihui Fong[1], Daisuke Sato[2], Colleen E. Clancy[1], Nicholas C. Vierra[1], James S. Trimmer[1], and L. Fernando Santana[1]

[1]Department of Physiology and Membrane Biology, School of Medicine, University of California, Davis, CA, USA

[2]Department of Pharmacology, School of Medicine, University of California, Davis, CA, USA

*Corresponding author. Email: lfsantana@ucdavis.edu

**This PDF file includes:**

Supplemental figures 1-4

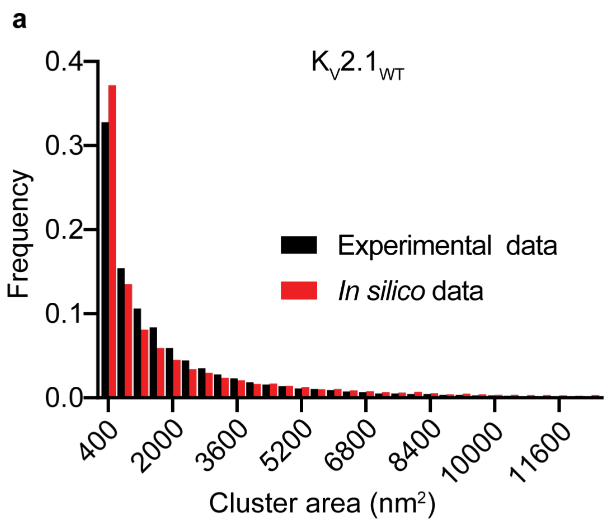


c

| Channel | $P_n$ | $P_g$ | $P_R$ | Mean experimental cluster size ($nm^2$) | Mean *in silico* cluster size ($nm^2$) | Mean experimental cluster density (clusters/$\mu m^2$) | Mean *in silico* cluster density (clusters/$\mu m^2$) |
|---|---|---|---|---|---|---|---|
| $K_V2.1_{WT}$ | 0.00050303 | 0.16532 | 0.001 | 2634 ± 143 | 2634.1 | 41 ± 4.7 | 40.389 |
| $K_V2.1_{S586A}$ | 0.000098767 | 0.050355 | 0.001 | 1860 ±104 | 1867.3 | 29 ± 3.7 | 28.758 |

**Supplemental Figure 1: The distributions of $K_V2.1_{WT}$ and $K_V2.1_{S586A}$ in HEK293T cells could be explained by a stochastic self-assembly mechanism.** (**a**) Histograms of experimental (black bars) and simulated (red bars) cluster area distributions as a relative frequency of $K_V2.1_{WT}$ in HEK293T cells. (**b**) Histograms of experimental (black bars) and simulated (red bars) cluster area distributions as a relative frequency of $K_V2.1_{S586A}$ in HEK293T cells. (**c**) Summary of experimental (n = 9 $K_V2.1_{WT}$ cells and 8 $K_V2.1_{S586A}$ cells) and *in silico* data. Probabilities of nucleation ($P_n$), growth ($P_g$) and removal ($P_R$) are presented.

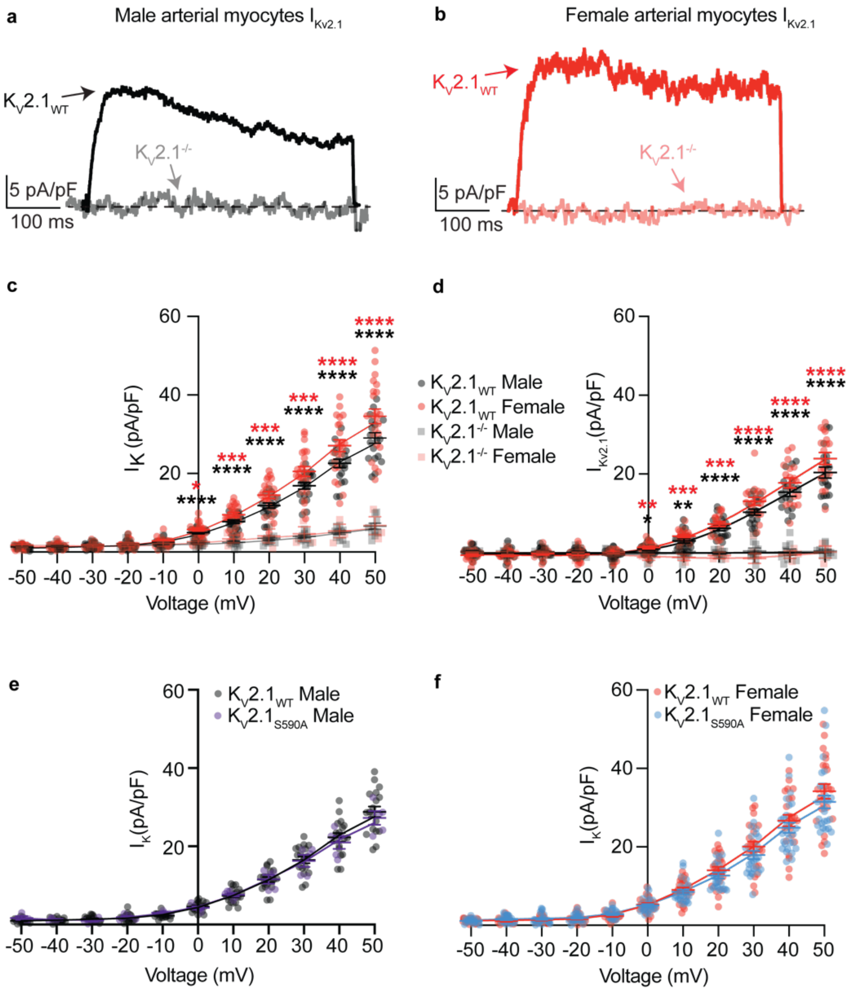


**Supplemental Figure 2: K$^+$ currents in $K_V2.1_{WT}$, $K_V2.1^{-/-}$ null and $K_V2.1_{S590A}$ myocytes.**
(**a**) Representative RY785-sensitive traces at +50 mV from $K_V2.1_{WT}$ (black) and $K_V2.1^{-/-}$ null (gray) male myocytes. (**b**) Representative traces at +50 mV from $K_V2.1_{WT}$ (red) and $K_V2.1^{-/-}$ null (pink) female myocytes. (**c**) IV relationship of total K$^+$ current ($I_K$) recorded from $K_V2.1_{WT}$ male (black), $K_V2.1^{-/-}$ null male (gray), $K_V2.1_{WT}$ female (red), and $K_V2.1^{-/-}$ null female (pink) myocytes. (**d**) IV relationship of RY785-sensitive ($K_V2.1$) currents recorded from $K_V2.1_{WT}$ male (black), $K_V2.1^{-/-}$ null male (gray), $K_V2.1_{WT}$ female (red), and $K_V2.1^{-/-}$ null female (pink) myocytes (n = 18 $K_V2.1_{WT}$ male, 13 $K_V2.1^{-/-}$ null male, 18 $K_V2.1_{WT}$ female, and 3 $K_V2.1^{-/-}$ null female myocytes). Black asterisks denote significance of comparisons between $K_V2.1_{WT}$ male and $K_V2.1^{-/-}$ null male myocytes and red asterisks denote significance of comparisons between $K_V2.1_{WT}$ female and $K_V2.1^{-/-}$ null female myocytes for panels **c** and **d**. (**e**) IV relationship of total K$^+$ current ($I_K$) recorded from $K_V2.1_{WT}$ male (black) and $K_V2.1_{S590A}$ male (purple) myocytes. (**f**) IV relationship of total K$^+$ current ($I_K$) recorded from $K_V2.1_{WT}$ female (red) and $K_V2.1_{S590A}$ female (blue) myocytes (n = 18 $K_V2.1_{WT}$ male, 11 $K_V2.1_{S590A}$ male, 30 $K_V2.1_{WT}$ female and 26 $K_V2.1_{S590A}$ female myocytes). *$P<0.05$, **$P<0.01$, ***$P<0.001$, ****$P<0.0001$. Error bars indicate mean ± SEM.

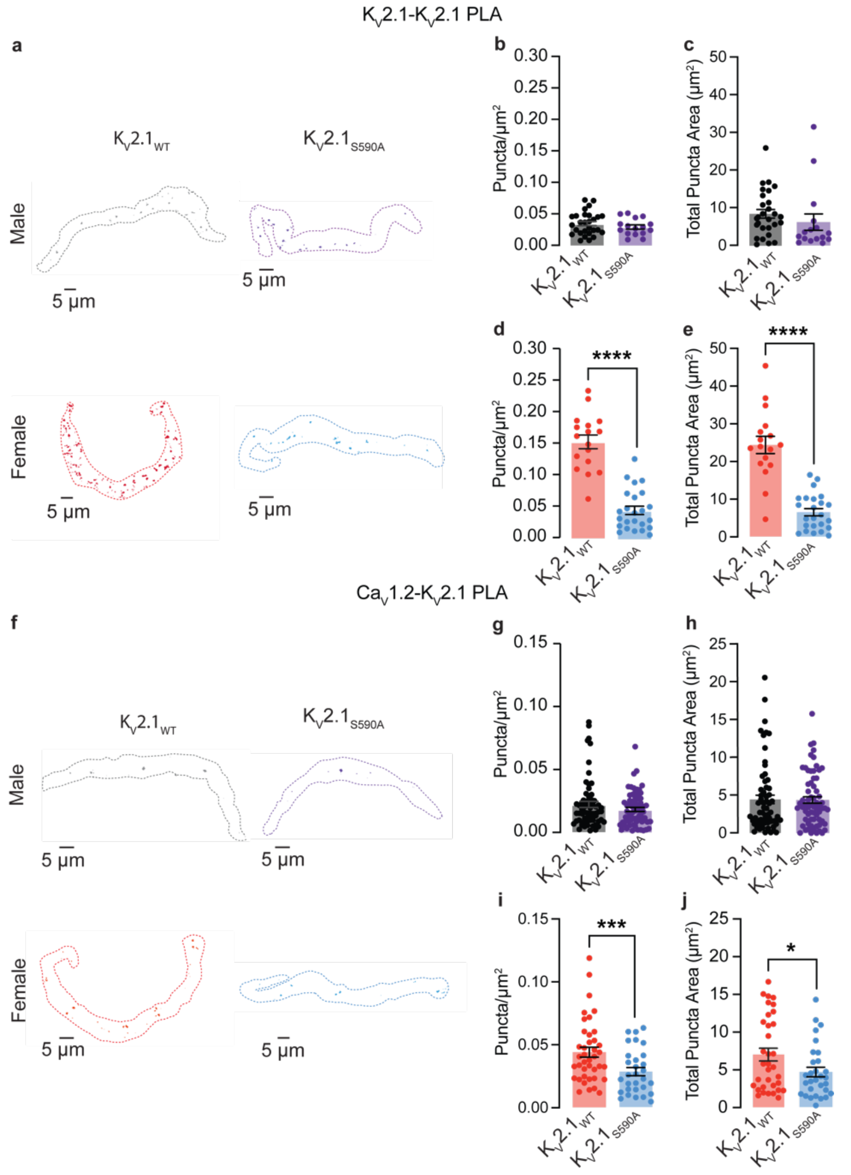


**Supplemental Figure 3: $K_V$2.1 and $Ca_V$1.2 proximity is decreased in female $K_V2.1_{S590A}$ myocytes.** (**a**) PLA images of representative $K_V$2.1-$K_V$2.1 channel proximity in arterial myocytes. Quantification of $K_V$2.1-$K_V$2.1 PLA puncta/µm$^2$ (**b**) and total $K_V$2.1-$K_V$2.1 PLA puncta area/cell (**c**) in male myocytes (n = 28 $K_V2.1_{WT}$ male, 16 $K_V2.1_{S590A}$ male myocytes). Quantification of $K_V$2.1-$K_V$2.1 PLA puncta/µm$^2$ (**d**) and total $K_V$2.1-$K_V$2.1 PLA puncta area/cell (**e**) in female myocytes (n = 17 $K_V2.1_{WT}$ female and 24 $K_V2.1_{S590A}$ female myocytes). (**f**) PLA images of representative $Ca_V$1.2-$K_V$2.1 channel proximity in arterial myocytes. Quantification of $Ca_V$1.2-$K_V$2.1 PLA puncta/µm$^2$ (**g**) and total $Ca_V$1.2-$K_V$2.1 PLA puncta area/cell (**h**) in male myocytes (n = 65 $K_V2.1_{WT}$ male, 66 $K_V2.1_{S590A}$ male myocytes). Quantification of $Ca_V$1.2-$K_V$2.1 PLA puncta/µm$^2$ (**i**) and total $Ca_V$1.2-$K_V$2.1 PLA puncta area/cell (**j**) in female myocytes (n = 40 $K_V2.1_{WT}$ female cells and 30 $K_V2.1_{S590A}$ female myocytes). *P<0.05, **P<0.01, ***P<0.001, ****P<0.0001. Error bars indicate mean ± SEM.

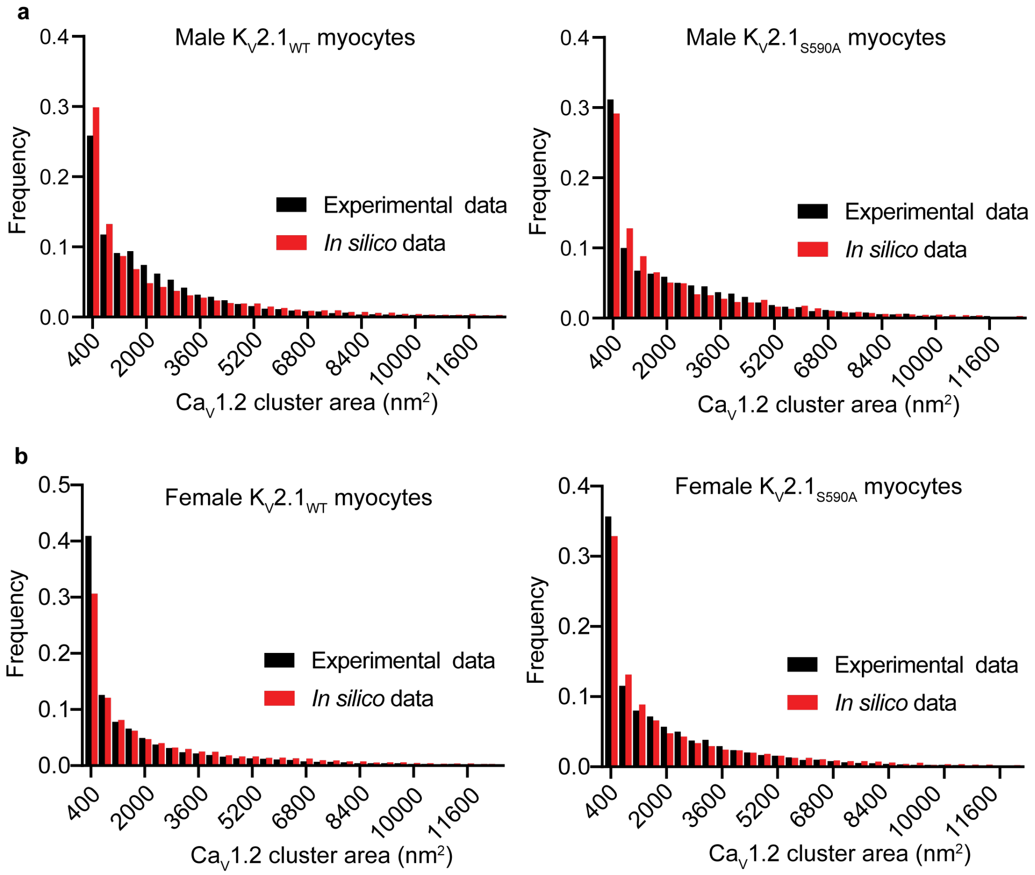


c

| Arterial Myocyte CaV1.2 | $P_n$ | $P_g$ | $P_R$ | Mean experimental cluster size (nm$^2$) | Mean *in silico* cluster size (nm$^2$) | Mean experimental cluster density (clusters/µm$^2$) | Mean *in silico* cluster density (clusters/µm$^2$) |
|---|---|---|---|---|---|---|---|
| WT male | 0.000056159 | 0.083203 | 0.001 | 2249 ± 54.53 | 2567.3 | 14 ± 1.2 | 14.062 |
| $K_V2.1_{S590A}$ male | 0.000047767 | 0.086086 | 0.001 | 2345 ± 81.84 | 2574.6 | 12 ± 2.2 | 12.176 |
| WT female | 0.000047826 | 0.10793 | 0.001 | 3098 ±163.8 | 2989.9 | 11 ± 1.1 | 10.881 |
| $K_V2.1_{S590A}$ female | 0.000043453 | 0.067511 | 0.001 | 2175 ± 91.72 | 2327.7 | 13 ± 1.8 | 13.32 |

**Supplemental Figure 4: The distributions of $K_V2.1_{WT}$ and $K_V2.1_{S590A}$ in arterial myocytes could be explained by a stochastic self-assembly mechanism. (a)** Histograms of experimental (black bars) and simulated (red bars) $Ca_V1.2$ cluster area distributions as a relative frequency from $K_V2.1_{WT}$ and $K_V2.1_{S590A}$ male arterial myocytes. (**b**) Histograms of experimental (black bars) and simulated (red bars) $Ca_V1.2$ cluster area distributions as a relative frequency from $K_V2.1_{WT}$ and $K_V2.1_{S590A}$ female arterial myocytes. (**c**) Summary of experimental (n = 13 $K_V2.1_{WT}$ male, 14 $K_V2.1_{S590A}$ male, 12 $K_V2.1_{WT}$ female, and 9 $K_V2.1_{S590A}$ female myocytes) and *in silico* data. Probabilities of nucleation ($P_n$), growth ($P_g$) and removal ($P_R$) are presented.
