# Supplementary material for: The formation of KV2.1 macro-clusters is required for sex-specific differences in L-type CaV1.2 clustering and function in arterial myocytes
Source: Commun Biol. 2023 Nov 14;6:1165. doi: 10.1038/s42003-023-05527-1 (PMC10645748; doi:10.1038/s42003-023-05527-1)
Supplement: Supplementary file 5 — Reporting Summary [file 42003_2023_5527_MOESM5_ESM.pdf]

nature portfolio

Corresponding author(s): Luis Fernando Santana

Last updated by author(s): October 27, 2023

Reporting Summary

Nature Portfolio wishes to improve the reproducibility of the work that we publish. This form provides structure for consistency and transparency in reporting. For further information on Nature Portfolio policies, see our Editorial Policies and the Editorial Policy Checklist.

Please do not complete any field with "not applicable" or n/a. Refer to the help text for what text to use if an item is not relevant to your study.
For final submission: please carefully check your responses for accuracy; you will not be able to make changes later.

Statistics

For all statistical analyses, confirm that the following items are present in the figure legend, table legend, main text, or Methods section.

n/a	Confirmed	
☐	☒	The exact sample size (*n*) for each experimental group/condition, given as a discrete number and unit of measurement
☐	☒	A statement on whether measurements were taken from distinct samples or whether the same sample was measured repeatedly
☐	☒	The statistical test(s) used AND whether they are one- or two-sided<br>*Only common tests should be described solely by name; describe more complex techniques in the Methods section.*
☒	☐	A description of all covariates tested
☐	☒	A description of any assumptions or corrections, such as tests of normality and adjustment for multiple comparisons
☐	☒	A full description of the statistical parameters including central tendency (e.g. means) or other basic estimates (e.g. regression coefficient) AND variation (e.g. standard deviation) or associated estimates of uncertainty (e.g. confidence intervals)
☐	☒	For null hypothesis testing, the test statistic (e.g. F, t, r) with confidence intervals, effect sizes, degrees of freedom and P value noted<br>*Give P values as exact values whenever suitable.*
☒	☐	For Bayesian analysis, information on the choice of priors and Markov chain Monte Carlo settings
☒	☐	For hierarchical and complex designs, identification of the appropriate level for tests and full reporting of outcomes
☒	☐	Estimates of effect sizes (e.g. Cohen's d, Pearson's r), indicating how they were calculated

Our web collection on statistics for biologists contains articles on many of the points above.

Software and code

Policy information about availability of computer code

Data collection	Quantitative PCR data was aquired using QuantStudio 7 Pro Real-time PCR System (Applied Biosystems).<br>Immunofluorescence images were collected using Fluoview FV300 (Olympus) or Dragonfly 2000 spinning disk (Andor).<br>Super-resolution images were collected using a DMI6000B TIRF Microscope (Leica)<br>Sparklet and split-Venus imaging was collected using an IX-70 inverted microscope in TIRF mode (Olympus).<br>Patch-clamp electrophysiology traces were recording using an Axopatch 200B amplifier and Digidata 1440 digitizer (Molecular Devices).
Data analysis	Data was analyzed using GraphPad Prism 10 software, Imaris 10 (Andor), Image J (Fiji) and pClamp 10 (Molecular Devices).

For manuscripts utilizing custom algorithms or software that are central to the research but not yet described in published literature, software must be made available to editors and reviewers. We strongly encourage code deposition in a community repository (e.g. GitHub). See the Nature Portfolio guidelines for submitting code & software for further information.

Data

Policy information about availability of data

All manuscripts must include a data availability statement. This statement should provide the following information, where applicable:

- Accession codes, unique identifiers, or web links for publicly available datasets
- A description of any restrictions on data availability
- For clinical datasets or third party data, please ensure that the statement adheres to our policy

All data are available in the main text or the supplementary materials.

Human research participants

Policy information about studies involving human research participants and Sex and Gender in Research.

Reporting on sex and gender	This study did not involve human research participants.
Population characteristics	This study did not involve human research participants.
Recruitment	This study did not involve human research participants.
Ethics oversight	This study did not involve human research participants.

Note that full information on the approval of the study protocol must also be provided in the manuscript.

Field-specific reporting

Please select the one below that is the best fit for your research. If you are not sure, read the appropriate sections before making your selection.

☒ Life sciences ☐ Behavioural & social sciences ☐ Ecological, evolutionary & environmental sciences

For a reference copy of the document with all sections, see nature.com/documents/nr-reporting-summary-flat.pdf

Life sciences study design

All studies must disclose on these points even when the disclosure is negative.

Sample size	Sample size was determined using a power analysis.
Data exclusions	No data were excluded.
Replication	Data was replicated in either technical or biological replicates.
Randomization	Mice were randomly selected for all experiments.
Blinding	Blinding was not necessary for our study.

Reporting for specific materials, systems and methods

We require information from authors about some types of materials, experimental systems and methods used in many studies. Here, indicate whether each material, system or method listed is relevant to your study. If you are not sure if a list item applies to your research, read the appropriate section before selecting a response.

Materials & experimental systems

n/a	Involved in the study
☐	☒ Antibodies
☐	☒ Eukaryotic cell lines
☒	☐ Palaeontology and archaeology
☐	☒ Animals and other organisms
☒	☐ Clinical data
☒	☐ Dual use research of concern

Methods

n/a	Involved in the study
☒	☐ ChIP-seq
☒	☐ Flow cytometry
☒	☐ MRI-based neuroimaging

Antibodies

Antibodies used	mouse anti-Kv2.1 K89/34; RRID: AB_2877280; NeuroMab, Davis, Ca<br>mouse anti-CaV1.2 L57/23; RRID: AB_2802123; NeuroMab, Davis, Ca<br>mouse anti-Kv2.1 pS590 phosphospecific mAb L100/1, Trimmer laboratory RRID:AB_2531886, Davis, Ca<br>rabbit anti-Kv2.1 KC; Trimmer laboratory, RRID:AB_2315767, Davis, Ca<br>Alexa Fluor 568-conjugated goat anti-mouse IgG, Molecular Probe<br>Alexa Fluor 647-conjugated donkey anti-rabbit IgG, Molecular Probes<br>Alexa Fluor 647-conjugated donkey anti-mouse IgG, Molecular Probes
Validation	Antibodies were validated as described by manufacturer's websites and previous studies.

Eukaryotic cell lines

Policy information about cell lines and Sex and Gender in Research

Cell line source(s)	HEK293T cells
Authentication	HEK293T cells sourced from ATCC
Mycoplasma contamination	Cell lines were not tested for mycoplasma contamination
Commonly misidentified lines (See ICLAC register)	No commonly misidentified lines were used.

Animals and other research organisms

Policy information about studies involving animals; ARRIVE guidelines recommended for reporting animal research, and Sex and Gender in Research

Laboratory animals	C57BL/6J (RRID: IMSR_JAX000664
Wild animals	Study did not involve wild animals
Reporting on sex	Study conducted sex-based analysis.
Field-collected samples	Study did not involve animals collected from the field.
Ethics oversight	All experiments were conducted in accordance with the University of California Institutional Animal Care and Use Committee guidelines.

Note that full information on the approval of the study protocol must also be provided in the manuscript.

This checklist template is licensed under a Creative Commons Attribution 4.0 International License, which permits use, sharing, adaptation, distribution and reproduction in any medium or format, as long as you give appropriate credit to the original author(s) and the source, provide a link to the Creative Commons license, and indicate if changes were made. The images or other third party material in this article are included in the article's Creative Commons license, unless indicated otherwise in a credit line to the material. If material is not included in the article's Creative Commons license and your intended use is not permitted by statutory regulation or exceeds the permitted use, you will need to obtain permission directly from the copyright holder. To view a copy of this license, visit http://creativecommons.org/licenses/by/4.0/
